# Dual-crosslinked hyaluronan hydrogels with rapid gelation and high injectability for stem cell protection

**DOI:** 10.1038/s41598-020-71462-4

**Published:** 2020-09-14

**Authors:** Chenggang Han, Hua Zhang, Yidong Wu, Xiuchao He, Xianwu Chen

**Affiliations:** 1 The Affiliated Hospital of Medical School, Ningbo University, Ningbo, 315020 China; 2 School of Materials Science and Engineering, Xi’an University of Technology, Xi’an, 710048 China; 3 Ningbo Institute of Materials Technology and Engineering, Chinese Academy of Science, Ningbo, 315201 China; 4 Li Huili Hospital Affiliate to Medicine of Ningbo University, Ningbo, 315041 China

**Keywords:** Stem cells, Materials science

## Abstract

Injectable dynamic hydrogels play a key role in cell transplantation to protect the cells from shear stress during injection. However, it still remains challenging to design dynamic hydrogels with fast gelation and high stability for protecting cells under flow due to the slow formation and exchange of most dynamic bonds. Here, a novel dual-crosslinked hydrogel system with fast dynamic crosslinks is developed by using methacrylate chitosan (CHMA) and aldehyde functionalized hyaluronate (oxidized HA, OHA). Based on the cooperation of electrostatic interaction between cationic amino of chitosan and anionic carboxyl of HA and Schiff-based crosslinking through amino and aldehyde groups, the dynamic CHMA-OHA hydrogel shows rapid gelation and high injectability. Further, the CHMA-OHA hydrogel is photopolymerized for achieving a high modulus and stability. Importantly, such hydrogels loaded with stem cells remains a cell viability (~ 92%) after extrusion. These results indicate that the CHMA-OHA hydrogel is a promising tissue engineering biomaterial for therapeutic cell delivery and 3D printing of encapsulated cell scaffolds.

## Introduction

Hyaluronic acid (HA) is a natural polysaccharide that is abundant in cartilage and skin, which plays a key structural role in the organization of the extracellular matrix as an organizing structure for the assembly of a proteoglycan^1,2^. Viscous solutions of high molecular-weight HA and its derivatives have been used in therapy for promoting wound healing in various tissues, such as a surgical aid in eye and skin. However, these solution systems are limited in application due to undesirable loss of material from the injection site and minimal control over important material properties (e.g., mechanics and degradation)^2^.

To address this issue, injectable HA hydrogels have been extensively developed for surgical implantation to fit variable target sites in patients using minimally invasive methods^3–5^. Numerous chemical crosslinking mechanisms have been explored to build injectable HA-based hydrogels, such as dynamic-chemistry, physical-assembly, host–guest interaction, and electrostatic interaction, etc^6^. Dynamic-chemistry (eg. acylhydrazone, Schiff-based bond etc.) provides an efficient, biocompatible strategy for bio-conjugation. It can be tuned to be dynamically covalent depending on the chemical structures^7–10^. This mechanism makes the hydrogels shear-thinning and self-healing. However, most of these dynamic progresses showed a slow formation and exchange, resulting in a high shear force during extrusion procedures. Such high shear stress leads to a deficit of live cells, with viabilities as low as 1–32% post-culture^11,12^. Although the dynamic gelation process can be accelerated by increasing polymer concentration^13^, adding catalyst^11^ or tuning pH^14^, these methods compromised the biocompatibility of the hydrogels. Therefore, it remains a challenge to achieve a rapid gelation for cell-shielding ability and good biocompatibility.

On the other hand, single dynamic HA hydrogel is inherently limited by low mechanical strength and rapid degradation^15–17^. To address these limitations, dual-crosslinking systems including non-covalent and covalent chemistries have provided an efficient approach to modulate the injectability, self-healing, degradation and mechanical strength^18–23^. For example, Heilshorn et al. designed an injectable hydrogel by two different physical crosslinking mechanisms including peptide-based molecular-recognition ex vivo and thermoresponsive crosslinkings^18,19^. Taking advantages of such two distinct crosslinking mechanisms, the hydrogel protected cells from shear force during syringe-needle flow and supported them high retention after injection. Burdick et al. developed a shear-thinning and rapid self-healing Dock-and-Lock (DL) hydrogel crosslinked by the molecular recognition and covalent interactions^20^. Upon a secondary light-initiated radical polymerization, the modulus of physically crosslinked DL hydrogels can be further 10-fold improved and their erosion rate drastically decreased. They also established a double-network hydrogel by supramolecular guest-host assembly for cell encapsulation and secondarily formed covalent crosslinks that achieved the elastic moduli ranging from 2.2 ± 0.2 kPa to 85.7 ± 4.5 kPa and improved the retention time in vivo^21,22^. Therefore, the dual cross-linking design is effective at increasing material mechanical strength and stability.

**Figure 1:**
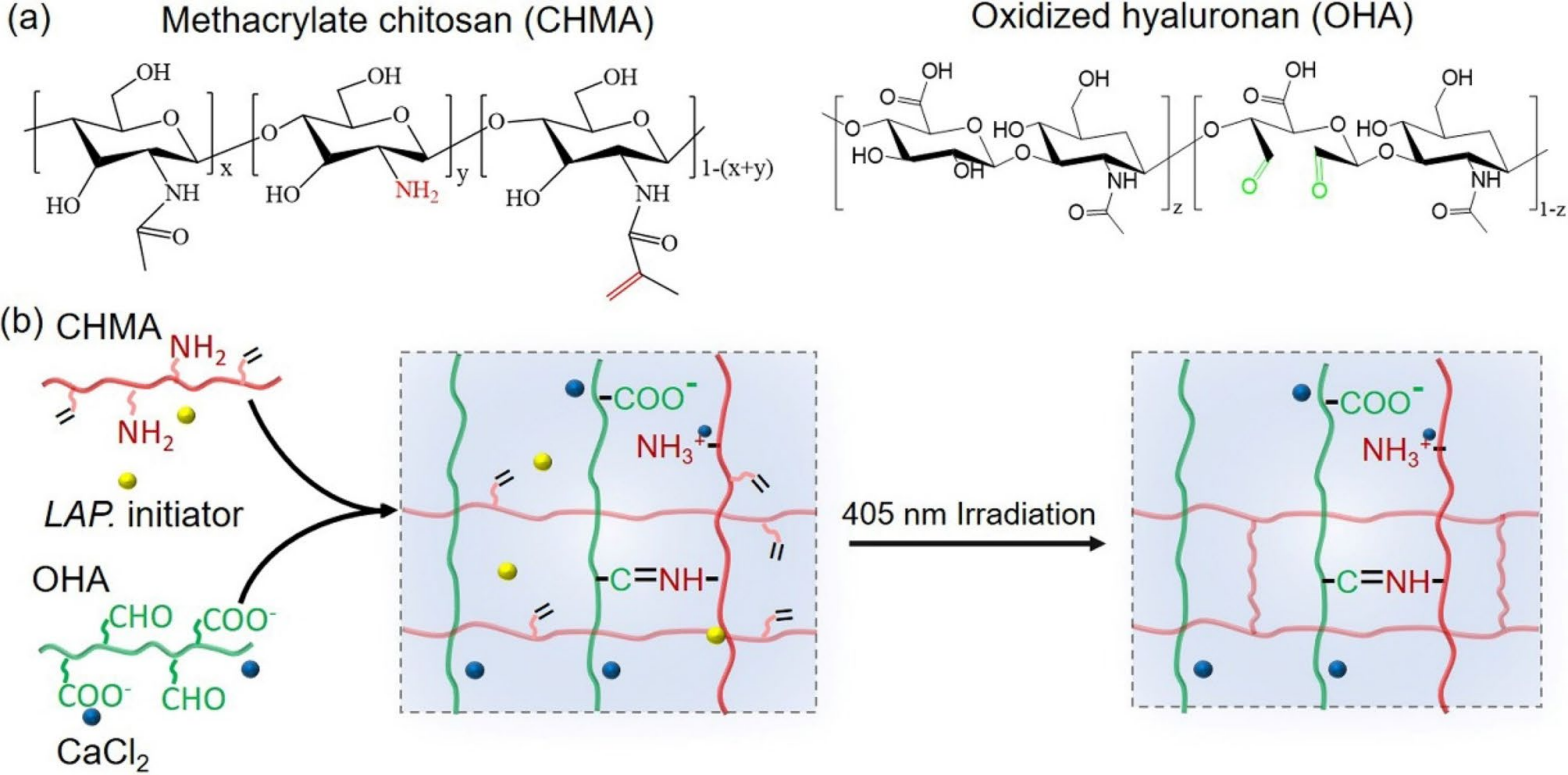
(**a**) Chemical structures of methacrylate chitosan (CHMA) and oxidized hyaluronic acid (OHA). (**b**) Schematic illustration of CHMA and OHA to facilitate the hydrogel formation by dynamic crosslinking and covalent crosslinking.

In this work, a novel dual-crosslinked hydrogel system with fast dynamic crosslinks was developed by using methacrylate chitosan (CHMA) and aldehyde functionalized HA (oxidized HA, OHA) (Fig. 1a). Due to electrostatic interactions between cationic amino of CHMA and anionic carboxyl of OHA and Schiff-based crosslinkings through amino and aldehyde groups, the CHMA and OHA first undergo rapid dynamic gelation (Fig. 1b). This mechanism make them shear-injecting and rapidly self-healing. After injection, a secondary covalent crosslinking occurs in situ via photopolymerization of methacrylates to stabilize the network and modulates the moduli as high as ∼60 times larger than moduli of gels based on dynamic crosslinking alone. More importantly, such CHMA-OHA hydrogel retained a high viability of above 92% after extrusion, making them promising for cell transformation and tissue engineering applications.

## Materials and methods

### Materials

Chitosan (CS, viscosity: 100 ~ 200 mPa·s, degree of deacetylation ~ 95%), methacrylic anhydride (MA, 94%), acetic acid, and dialysis tubing with molecular weight cut off range 8,000–14,000 were purchased from Sinopharm Chemical Reagent Co. Methacrylate chitosan (CHMA) was synthesized by single-step chemoselective N-acylation reaction between amino group and methacrylic anhydride according to previous reports^24,25^. ^1^H-NMR confirmed that the degree of substitution is  ~ 22.6% under experiment conditions. Hyaluronic acid (HA, 500-700 kDA) and sodium periodate were purchased from Shanghai Aladdin Bio-Chem Technology Co. Lithium phenyl-2, 4, 6-trimethylbenzoylphosphinate (LAP) photoinitiator was supplied by.Tokyo Chemical Industry Co., Ltd.

### Synthesis of oxidized hyaluronic acid

Oxidized hyaluronic acid (OHA) was synthesized by open-ringing reaction using sodium periodate as oxidant according to the previous reports^8^. Briefly, 1.0 g of sodium hyaluronate was dissolved completely in 100 mL of double distilled water at a concentration of 10 mg/mL. Sodium periodate aqueous solution (0.1 g dissolved in 5 mL water) was subsequently added into the solution. The mixture was stirred for 3 h at room temperature in the dark, followed by adding 0.1 mL of ethylene glycol for another 1 h reaction to inactivate any unreacted periodate. The resulting solution was purified by exhaustive dialysis (MW cutoff 3500) against water for one week, and then was fully freeze-dried at − 65 °C. The purified OHA was stored at − 80 °C for further use.

### CHMA-OHA hydrogel formation

A 1.5 wt% CHMA solution was prepared by dissolving CHMA in water containing 100 mM/L CaCl_2_ as electrostatic shielding agent and 0.1 wt% lithium phenyl-2, 4, 6-trimethylbenzoylphosphinate (LAP) photoinitiator. 6 wt% OHA solution was prepared by dissolving OHA in water. CHMA and OHA solutions with 1:1 were vigorously stirred using the pipet tip. The resulting hydrogel was further exposed to 405 nm blue light (10 mW/cm^2^) for 120 s.

### Characterization

#### Gel permeation chromatography

A Verotech PL-GPC 50 system (Agilent Technologies, USA) was applied to trace possible changes in molecular weight (Mw) and polydispersity index (PDI). The HA and OHA were dissolved separately in deionized water with a concentration of 2.5 mg/ml. The solutions were then filtered by a 0.22 μm membrane to avoid plugging of the columns. The samples were injected with a PL-AS RT Autosampler for PL-GPC 50 and deionized water was used as the mobile phase (1 mL/min, 35 °C). The calibration was created using polystyrene standards with a narrow molecular weight distribution.

#### Characterization of OHA by hydroxylamine hydrochloride titration assay

The oxidation degree of HA was determined by measuring the aldehyde content via hydroxylamine hydrochloride titration method as previously described^26,27^. Briefly, 0.1g of lyophilized OHA was dissolved in 25 mL of hydroxylamine hydrochloride solution (0.25 mol/L, pH = 4.5) containing 0.0003 wt % of methyl orange reagent, and stirred for 24 h at room temperature. The conversion of aldehydes into oximes was followed by titration of the released hydrochloric acid with 0.1 mol/L of sodium hydroxide solution until the red-to-yellow end point (pH≈5.0) was achieved. The change of pH with the volume of added sodium hydroxide solution was recorded by using a pH meter. The actual oxidization degree of the OHA was the average of three experiments. The related reactions and calculation formula are as follows: 

1 $$ {\text{HA}}-({\text{CHO}}){\text{n}} + {\text{n}} {\text{H}}_{2}N-{\text{OH}}{\cdot}{\text{HCl}} = {\text{HA}}-({\text{CH}}={\text{N}}-{\text{OH}}){\text{n}} + {\text{n}} {\text{H}}_{2}{\text{O}} + {\text{nHCl}} $$

                                                                                                                                                                                                             

2 $${\text{nHCl}} + {\text{NaOH}} = {\text{NaCl}} + {\text{H}}_{2}{\text{O}}$$

$$ {\text{Oxidation degree}} {\%} = 403 c \times \Delta V \times 10^{-3} /2w $$

where *c* is the concentration of sodium hydroxide solution in mol/L; Δ*V* is the consumed volume of sodium hydroxide solution in mL; *w* is the weight of HA-CHO in grams; and 403 is the molecular weight of saccharide repeating units in g/mol.

#### Rheology measurements

The rheological behaviors of CHMA-OHA hydrogel were assessed with time sweeps and shear strain sweeps using a DHR-2 rheometer (TA Instruments) with a quartz plate connected to a blue light source. A plate geometry with a solvent trap, 20 mm diameter, and 500 μm gap distance was used. CHMA-OHA hydrogels of specified compositions were formed by mixing together CHMA/OHA components, and loaded onto the rheometer. Time sweeps were performed for in-situ gelation observation. To measure the response of rheological properties to photopolymerization, in situ polymerization was performed with in-situ dynamic crosslinking with 405 nm wavelength irradiation at 10 mW cm^−2^ intensity using a dental lamp attached to a light guide for different formulations for 2 min via a light-curing stage during oscillatory time sweeps at a frequency of 10 rad/s and a strain of 0.5%. Experiments were repeated for a minimum of three times, and representative data was presented. Strain sweeps from 0.1% to 1,000% were performed for gel-sol transformation. Alternative strain sweeps with 1% low strain and 1,000% high strain were performed for shear recovery experiments.

#### Morphology observations

The specimens for field emission scanning electron microscope (SEM) were frozen at − 20 °C for 12 min and then lyophilized at − 65 °C for 72 h. Subsequently, the samples were fractured in liquid nitrogen. The cross-section of hydrogels was sputtered with gold for SEM imaging by using an SEM4800 (Hitachi, Tokyo, Japan) instrument at an accelerating voltage of 4.0 kV.

#### Swelling

The swelling properties of CHMA-OHA scaffolds were demonstrated by immersing the weighed freeze-dried scaffolds (Wd) in a large excess of water at 37 °C. At predetermined time, the samples were retrieved and gently blotted with filter paper to remove the excess of water lying on the surfaces. The wet weight of the CHMA-OHA hydrogels was determined using an electronic balance. As the weights of samples kept constant, the equilibrium of swelling was reached. All experiments were done in triplicate. The swelling ratio was defined as SR= (*W*_s_−*W*_d_)/*W*_d_. 

#### Stem cell 3D culture

Primary rat bone mesenchymal stem cells (BMSCs) supported by the medical school of Ningbo University were expanded in low-glucose Dubecco’s Modified Eagle Medium (DMEM, Gibco) supplemented with 10% fetal bovine serum (FBS, Gibco) and 1% penicillin/streptomycin (Gibco). The medium was changed every other day. The cells were passaged upon above 70% confluence and only four-passage BMSCs were used for all experimental studies.

For in vitro cell injection studies, trypsinized BMSCs with a cell density of 106 cells/mL were suspended in 1.5 wt% CHMA and 6 wt% OHA pre-polymer solutions containing specified concentrations of LAP and CaCl2. The cell-laden solutions were mixed at 1v:1v and placed in the barrel of a 1 mL syringe fitted with a 25-gauge needle. The mixture was allowed to gel for 5 min before injecting into a 24-well plate using a syringe pump (LSP02-2B; LONGER Instruments) at a flow rate of 0.1 mL/min. Cell viability was determined using LIVE/DEAD viability/cytotoxicity kit (Biovision) at 30 min post-injection and at days 1, 3, and 5 post-injection (n = 5), according to manufacturer's instructions. The live (calcein AM labeled) and dead (ethidium homodimer labeled) cells were visualized using a Laser Scanning Confocal Microscope (LSCM, Leica TCS-SP8, Germany). Viability is reported as the percentage of cells with positive calcein staining (n = 3).

#### Statistical analysis

All data obtained from each group were averaged and presented as mean ± standard deviation. For hydrogel swelling, values were compared between dynamic crosslinking and dual-crosslinking groups using a Student’s t-test. Cell viability from each group was compared by one-way analysis of variance (ANOVA) with Tukey post hoc test. Values were considered to be significantly different when the p value was <0.05.

## Results and discussion

### Synthesis and viscoelasticity of dual-crosslinked hydrogels

Partially oxidized hyaluronate (OHA) containing two aldehyde groups in the repeating unit of hyaluronate (HA) was prepared using sodium periodate as the oxidant. The chemical structure is presented in Figure 1a. The degree of oxidation is around 20% according to the hydroxylamine hydrochloride titration assay. The mass average molecular weight (Mw) of OHA is about 149 kDA, which is significantly lower than that of HA (544 kDA) (Figure S1, Supporting Information). This result is consistent with the previous study, suggesting that glycosidic bond was also disassociated in the oxidant^28^.

Dual-crosslinked CHMA-OHA hydrogels were prepare by a two-step crosslinking method. Firstly, the CHMA with 22.6% substitution and OHA chains were crosslinked by dynamic Schiff-based bonds and electrostatic interactions (Fig. 1b). The mixed CHMA-OHA gels can be rapid formed within 10 s (Movie S1, Supporting Information). The time sweep rheological measurement showed that the moduli of such dynamic hydrogel were nearly constant (G’: ~410 Pa, G’’: ~ 80 Pa), suggesting a superior gelation ability. Following by the photopolymerization, the moduli of the dynamic CHMA-OHA hydrogel abruptly increase and achieve the full-gelation within 60s. The plateau storage moduli of dual-crosslinked hydrogel were ~24 kPa, which is about 60 times comparing to the dynamic hydrogel.

**Figure 2:**
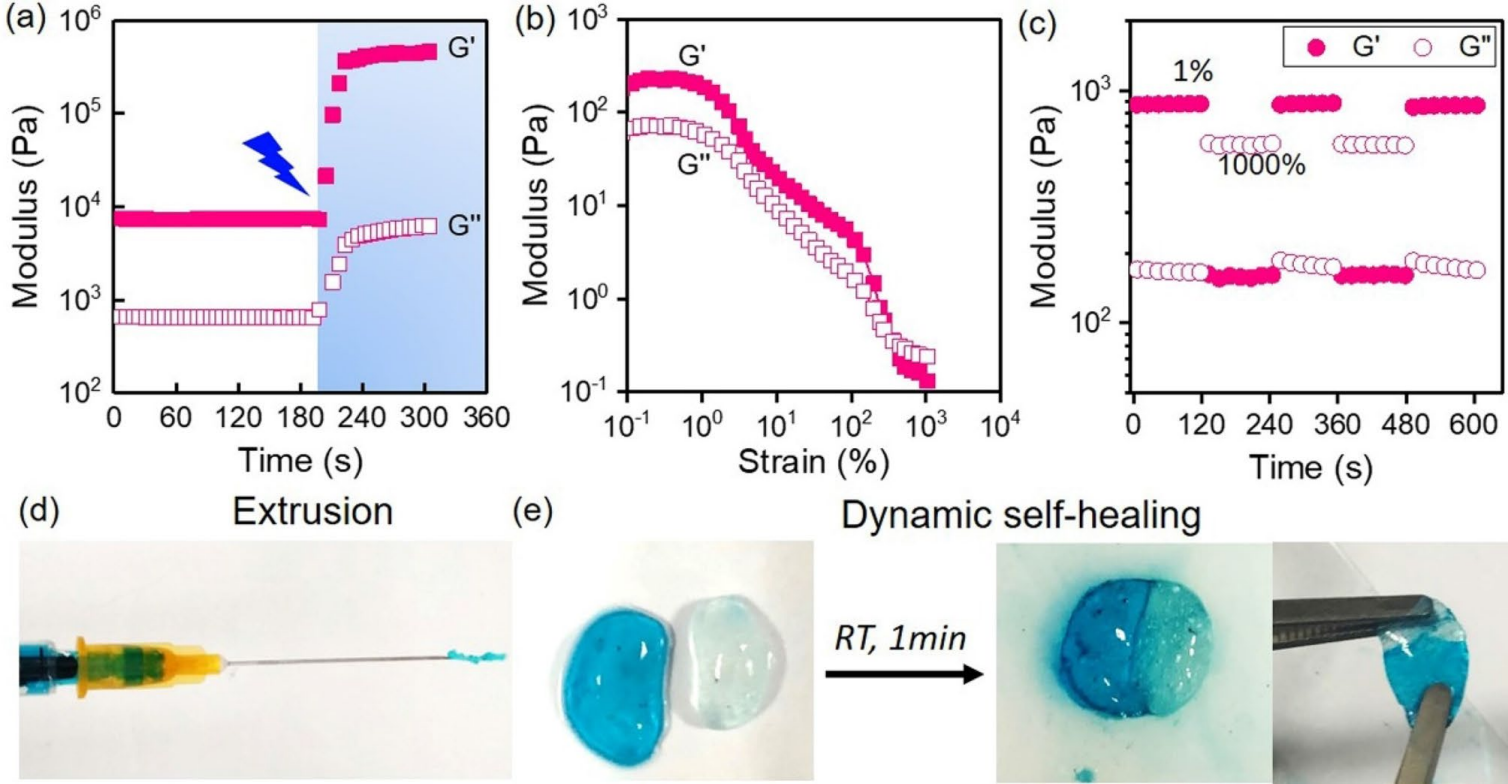
Rheological analysis of the CHMA-OHA hydrogel. (**a**) Real-time gelation observation including dynamic crosslinking and photopolymerization. (**b**) Representative strain sweep with G′ and G″ shows the shear yield of dynamic-hydrogel with increasing strain. (**c**) Corresponding recovery of dynamic hydrogel undergoing cyclic deformation of 1% and 1,000% strain at 10 rad/s with G′ and G″. (**d**) Photo of ejecting gels through a 25 G needle without clogging. (**e**) In situ self-healing macroscopic images of the dynamic CHMA-OHA hydrogel at room temperature.

Dynamic crosslinked CHMA-OHA hydrogels can return to a liquid state when applying an excessive shear to rupture dynamic crosslinks (Fig. 2b). As the shear strain was increased from 0.1% to above 1,000%, the modulus significantly decreased. When the strains were above 420%, the G′ was lower than the G″, representing a gel-sol transformation behavior. Moreover, such behavior was reversable. As shown in Fig. 2c, upon the removal of shear, dynamic crosslinks instantly reformed upon the removal of shear strain from 1000% to 1%, causing the material to self-heal (> 95%) within 10 s of removal of high-strain conditions. Therefore, such hydrogel can be ejected easily through the thin needle without clogging (Fig. 2d). Moreover, the completely separated dynamic hydrogels can rapidly self-heal and resist the mechanical stretch after 1 min of contacting at room temperature without any external assistance (Fig. 2e). Such rapid self-healing kinetics attribute to the inherently fast on-rate kinetics of Schiff-based bonds and electrostatic interactions. These results indicated that the dynamic crosslinked CHMA-OHA gels exhibit a good injectability, where they would become thin and flow as liquids under shear during delivery and rapidly re-assemble into hydrogels upon reaching the desired injection site in situ.

### Microstructures and swelling behaviors

**Figure 3:**
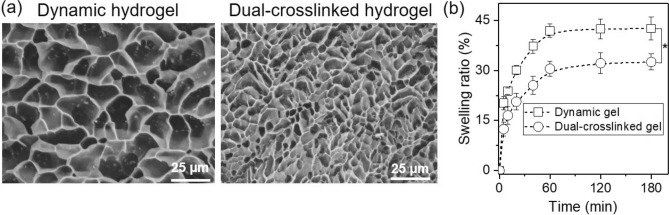
(**a**) SEM images of CHMA-OHA scaffolds. (**b**) Swelling behavior of CHMA-OHA hydrogels (*p < 0.05).

The microporous density and sizes of hydrogel directly reflect the crosslinking structure. As shown in Fig. 3a, both dynamic hydrogel and dual-crosslinked CHMA-OHA hydrogels both show a typical three-dimensional porous structure. However, comparing to the dynamic hydrogel, the dual-corsslinked CHMA-OHA hydrogel shows a highly dense porous structure. The pore diameters (~ 5 µm) were significantly lower than those of dynamic hydrogel (> 20 µm). These results indicated that dual-crosslinked hydrogel has a highly crosslinking network than dynamic hydrogel.

Such microstructure differences could be further reflected from the swelling characteristics of hydrogels. The swelling kinetic of the CHMA-OHA hydrogel was investigated in water at 37 °C. As shown in Fig. 3b, the swelling ratio increased with the hydration time up to 60 min. Obviously, when hydrogels were further crosslinked by a blue light irradiation, they exhibited a lower swelling ratio (~ 31%) than that of dynamic hydrogels (~ 42%). These results further corroborate the formation of the more interactions in the dual-crosslinked hydrogels.

### Stem cell protection within CHMA-OHA hydrogels

To determine the viability of stem cells loaded in the CHMA-OHA hydrogels, rat bone mesenchymal stem cells (BMSCs) were gently encapsulated into the hydrogels. The ydrogel was rapidly formed in the syringe and ejected through a 25 G syringe needle. The BMSCs-loaded hydrogels were further transferred into the culture medium for in vitro 3D incubating. The LIVE/DEAD assay was performed after 30 min to examine the effect of CHMA-OHA hydrogels on the protection of encapsulated cells during injection.

**Figure 4:**
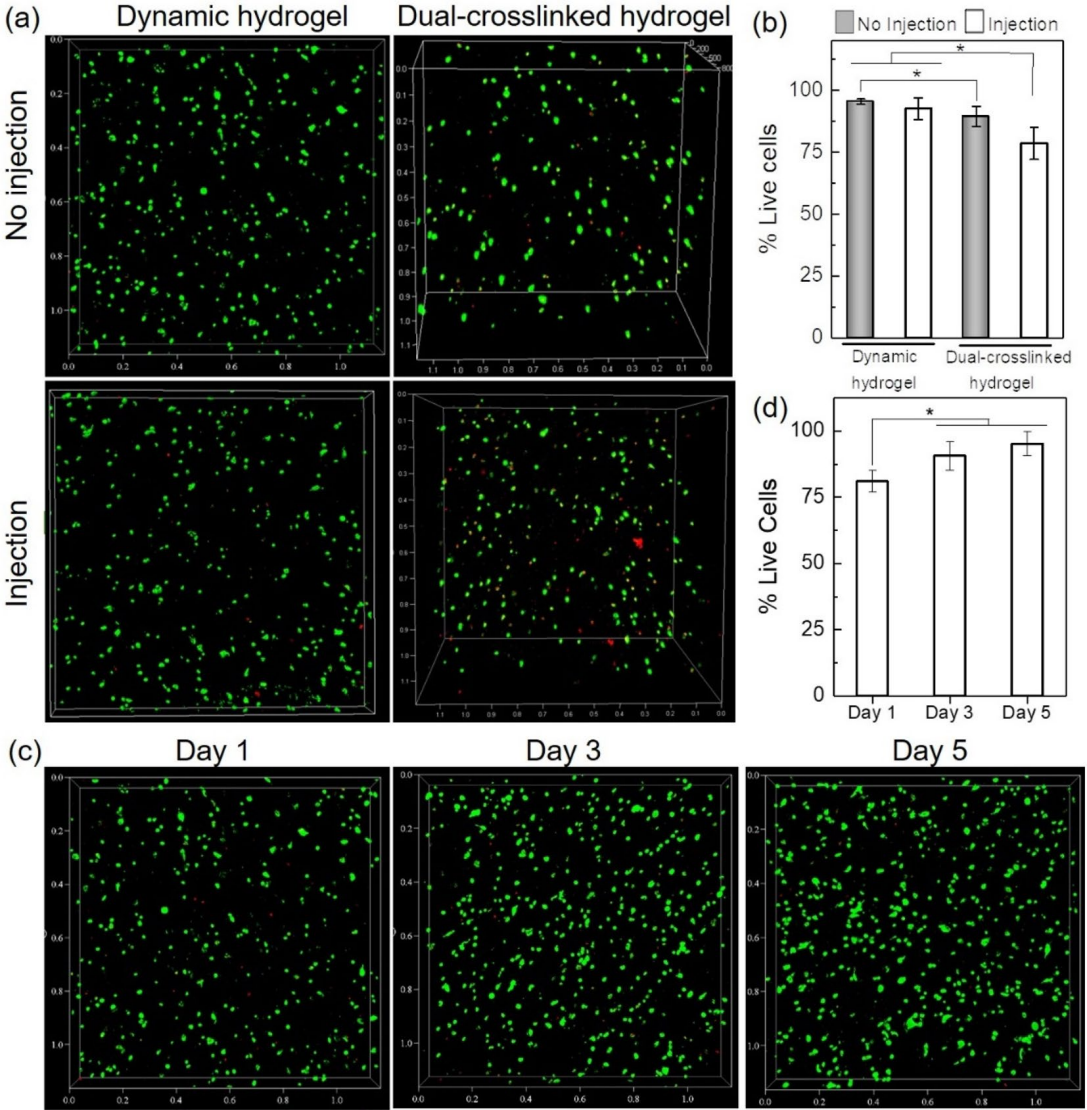
In vitro cytocompatibility of CHMA-OHA hydrogel. (**a**) LIVE/DEAD stain of encapsulated BMSCs in CHMA-OHA hydrogel without injection and with injection. (**b**) Quantification of cell viability from Live/DEAD assay (n = 3, *p < 0.05). (**c**) Live/Dead staining of the encapsulated BMSCs for 1, 3 and 5 days. (**d**) The long-term viability of cells for 1, 3 and 5 days (*n*=3, *p < 0.05).

As shown in Fig. 4, the BMSCs encapsulated in dynamic hydrogels exhibit a homogenous cell distribution and high viability of >95%. After injection, around 92% of the BMSCs were still alive within the hydrogel, statistically similar to the noninjected controls. These results suggested that the hydrogel provided significant cell protection from the damaging mechanical forces experienced during cell transplantation. When hydrogels were further crosslinked by UV-light irradiation (10 mW/cm^2^), more cells exhibit membrane damage, resulting in the decrease of cell viability with 89% and 78% for non-injected and post-injected gels, respectively. It is lower than those within dynamic hydrogels but much higher than many previous reports^29,30^. The BMSCs within post-injected hydrogels with a secondary network were further maintained for 1, 3 and 5 days to demonstrate the long-term viability of the encapsulated cells. Live/Dead results showed that the cells remained a round morphology and homogenous distribution (Fig. 4c). More importantly, they exhibit a high viability with the extension of incubation time. As shown in Fig. 4d, the cell viability remains over 80% on the first day. Subsequently, dead cells significantly decrease on the third day and the cell viability is over 90%. The viability of cells cultured on the fifth day is up to 95%. These results prove that the CHMA-OHA hydrogel system is a cyto-compatible and non-toxic matrix for supporting cell encapsulation, delivery by injection and cell proliferation in vitro.

## Conclusions

In summary, we have developed a new dual-crosslinked hydrogel system with rapid gelation dynamics, high injectability and rapid self-healing. Such hydrogel was prepared by using methacrylate chitosan (CHMA) and oxidized hyaluronate (OHA). Due to electrostatic interactions between cationic amino of chitosan and anionic carboxyl of HA and Schiff-based crosslinking through amino and aldehyde groups, the CHMA and OHA could rapidly form a dynamic hydrogel. Such dynamic hydrogel exhibited a gel-sol transformation above 420% strain and could self-recovery within only 10 s. More importantly, the CHMA-OHA hydrogel could significantly improve cell protection during injection. The cell viability kept around 92% in the face of injection. We envision that this novel dual-crosslinked hydrogel would be an attractive biomaterial for therapeutic cell delivery and 3D printing of encapsulated cell scaffolds.

## Supplementary information

Supplementary file1

Supplementary file2
