# Supplementary material for: Dual-crosslinked hyaluronan hydrogels with rapid gelation and high injectability for stem cell protection
Source: Sci Rep. 2020 Sep 14;10:14997. doi: 10.1038/s41598-020-71462-4 (PMC7490415; doi:10.1038/s41598-020-71462-4)
Supplement: Supplementary file 1 — Supplementary file1 [file 41598_2020_71462_MOESM1_ESM.docx]

**Supplementary information**

**Dual-crosslinked Hyaluronan Hydrogels with Rapid Gelation and High Injectability for Stem Cell Protection**

Chenggang Han^a^, Hua Zhang^b,c*^, Yidong Wu^d^, Xiuchao He^a^, Xianwu Chen^a*^

1. The Affiliated Hospital of Medical School, Ningbo University, Ningbo 315020, China.
2. School of Materials Science and Engineering, Xi’an University of Technology, Xi’an 710048, China.
3. Ningbo Institute of Materials Technology & Engineering, Chinese Academy of Science, Ningbo 315201, China.
4. Li Huili Hospital Affiliate to Medicine of Ningbo University, Ningbo 315041, China

E-mail: [zhanghua@nimte.ac.cn](mailto:zhanghua@nimte.ac.cn); [chenxianwu1982@163.com](mailto:chenxianwu1982@163.com)

Figure S1. The mass-averaged molecular weight (*M_w_*) and number-averaged molecular weight (*M_n_*) were determined by gel permeation chromatography. The reported values are *M_w_*.
